# Fewer complications after laparoscopic nephrectomy as compared to the open procedure with the modified Clavien classification system - a retrospective analysis from Southern China

**DOI:** 10.1186/1477-7819-12-242

**Published:** 2014-07-31

**Authors:** Hua Xu, Qiang Ding, Hao-wen Jiang

**Affiliations:** 1 Department of Urology, Huashan Hospital, Fudan University, 12 WuLuMuQi Middle Road, 200040 Shanghai, PR China

**Keywords:** Clavien classification system, Complication, Laparoscopy, Nephrectomy

## Abstract

**Background:**

The objective of the study is to compare complication rates of laparoscopic nephrectomy and open nephrectomy using a standardized classification method

**Methods:**

We retrospectively included 843 patients from March 2006 to November 2012, of whom 88 had laparoscopic radical nephrectomy (LRN), 526 had open radical nephrectomy (ORN), 42 had laparoscopic partial nephrectomy (LPN), and 187 had open partial nephrectomy (OPN). A modified Clavien classification system was applied to quantify complications of nephrectomy. Fisher’s exact or chi-square test were used to compare complication rates between laparoscopic and open approaches.

**Results:**

The overall complication rate was 19.31%, 30.04%, 35.71%, and 36.36% in LRN, ORN, LPN, and OPN, respectively. More Grade II complications (odds ratio = 2.593, 95% CI 1.172 to 5.737, *P* = 0.010) and longer postoperation hospital stay (9.2 days and 7.6 days, *P* < 0.001) were observed in ORN compared with LRN. In multivariable analysis, surgical approach (LRN/ORN) (*P* = 0.036), age (*P* = 0.044), height (*P* = 0.020), systolic pressure (*P* = 0.012), fasting blood glucose level (*P* = 0.032), and blood loss during operation (*P* = 0.011) were significant predictors for grade II complications in radical nephrectomy. LPN had similar complication rates compared with OPN.

**Conclusions:**

In conclusion, LRN had the advantages of less grade II complications and shorter postoperation hospital stay than ORN. Older age and more blood loss during operation would also contribute to more grade II complications in radical nephrectomy.

## Background

Renal tumor is one of the most frequently diagnosed urological tumors, ranking eighth among all the tumors in the United States in 2012 [1]. Laparoscopic technology was applied in urological surgeries soon after it was put in practice. In 1991, the first laparoscopic nephrectomy was performed by Clayman and colleagues [2]. Since then, a number of studies [3,4] have reported that laparoscopic surgery offers several advantages over traditional open surgery (such as alleviating postoperative pain, a decreased length of hospital stay and earlier recovery), while achieving equivalent cancer control.

Besides the long-term outcome of nephrectomy, the short-term postoperation outcome (mainly the complication rate) is also generally used to demonstrate the quality of care which has been provided to the patient. Several studies [4,5] reported that laparoscopic nephrectomy had similar complication rates to open nephrectomy. The Clavien classification system (CCS) has been proposed as a mean of quantifying the complications of surgery [6]. It has recently been modified and prospectively validated in a large patient cohort [7]. Few studies have applied the modified CCS in urological surgeries. Also, no study in China has investigated the complication rate of nephrectomy by using this standardized method. We conducted this study to investigate the risk of complications in laparoscopic nephrectomy and open nephrectomy in the Chinese population.

## Methods

### Patient source

After obtaining approval of the Institutional Review Board of Huashan Hospital, Fudan University, Shanghai China, a retrospective study of medical records from March 2006 to November 2012 was performed to identify all patients who underwent radical or partial nephrectomy for presumed renal tumor. After reviewing medical records and the patient information system, we identified a total of 843 patients with detailed admission information and clinical information, as well as operative, postoperative and pathological information. All subjects were ethnic Han Chinese. Among them, 614 received radical nephrectomy (88 had laparoscopic radical nephrectomy (LRN); 526 had open radical nephrectomy (ORN)) and 229 had partial nephrectomy (42 had laparoscopic partial nephrectomy (LPN); 187 had open partial nephrectomy (OPN)). Both open nephrectomy and laparoscopic nephrectomy have been performed for a long time in our hospital, open nephrectomy for more than 50 years. Our department was the first to perform laparoscopic surgeries in our hospital, and we have over 10 years experience performing laparoscopic nephrectomy. In order to reduce potential bias, all 843 patients we included received either radical nephrectomy or partial nephrectomy via the transretroperitoneal approach in the modified lateral decubitus position. Both LRN and LPN were performed with four trocars (anterior axillary line 2 cm above crista iliaca, mid-axillary line 2 cm above crista iliaca, anterior axillary line 2 cm under the costal margin, and posterior axillary line 2 cm under the costal margin). In LRN, the excised kidney was taken out through incision of the two holes above the crista iliaca. In LPN, the excised tumor was taken out through the hole of the mid-axillary line 2 cm above crista iliaca.

Written consent was given by the patients for their information to be stored in the hospital database and used for research.

### Patient characteristics

Patient characteristics were obtained mainly from electronic medical records in Huashan Hospital. Basic information, such as height, weight, blood pressure, fasting blood glucose level, medical history of hypertension and diabetes, were collected on admission. Diabetes mellitus was defined as pharmacological treatment for type 2 diabetes mellitus, elevated fasting plasma glucose level (≥7.0 mmol/L), or elevated 2-hour postprandial plasma glucose level (≥11.1 mmol/L) on admission. Hypertension was defined as pharmacological treatment for hypertension or elevated blood pressure (≥140/90 mmHg) on admission. Body mass index (BMI) was calculated as the ratio of the patient weight (kg) divided by the square of the patient height (m). Patients were divided into four groups based on the recommendation from the World Health Organization (WHO) [8] and Guidance of Obesity Control in China: underweight, <18.5 kg/m^2^; normal, 18.5 to 23.9 kg/m^2^; overweight, 24 to 27.9 kg/m^2^; obese, ≥28 kg/m^2^. Pathological tumor size was mainly determined by imaging. For those without detailed imaging information, the intra-operative size was used [9]. Pathological profiles were blindly determined by two independent pathologists according to the 2004 WHO classification [10]. Information about volume of blood loss and blood transfusion during operation were collected from operation notes. Length of postoperation stay was defined as the period from the day when nephrectomy was performed to the day of discharge. Length of operation time was defined as the period from the beginning of incision to the end of wound closure.

### Primary outcomes

The primary outcome that we assessed was post-nephrectomy complications of patients. Patients were followed-up until discharge. If patients were discharged earlier, they would be followed-up for 30 days postoperatively. Operative characteristics and complications were recorded in medical records. We identified specific International Statistical Classification of Diseases and Related Health Problems-9 codes for acute renal failure, cardiac complications, wound complications, postoperative infection, gastrointestinal complications, pulmonary complications, thromboembolic complications, postoperative hemorrhage, neurologic complications and miscellaneous technical complications related to surgery. Each measure has been described previously by the Complications Screening Program [11].

In order to quantify surgical complication severity, we applied the standardized method (modified CCS) 7 to divide the complications into five major grades: Grade I, any deviation from the normal intraoperative or postoperative course, including the need for pharmacologic treatment other than antiemetics, antipyretics, analgesics, diuretics, electrolytes, or physiotherapy; Grade II, complications needing only the use of intravenous medications, total intravenous nutrition, or blood transfusion; Grade IIIa, complications needing surgical, endoscopic, or radiologic intervention under local anesthesia; Grade IIIb, complications calling for surgical, endoscopic, or radiologic intervention under general anesthesia; Grade IVa, life-threatening complications requiring ICU management - single organ dysfunction; Grade IVb, life-threatening complications requiring ICU management - multi-organ dysfunction; Grade V, death of the patient (Table 1). Grade I and Grade II were defined as low grade, and Grade III, Grade IV, and Grade V were defined as high grade.

**Table 1:** Modified Clavien classification system for surgical complications

**Low grade**	
Grade I	Any deviation from the normal intraoperative or postoperative course, including the need for pharmacologic treatment other than antiemetics, antipyretics, analgesics, diuretics, electrolytes, or physiotherapy
Grade II	Complications needing only the use of intravenous medications, total intravenous nutrition, or blood transfusion
**High grade**	
Grade IIIa	Complications needing surgical, endoscopic, or radiologic intervention under local anesthesia
Grade IIIb	Complications needing surgical, endoscopic, or radiologic intervention under general anesthesia
Grade IVa	Life-threatening complications requiring ICU management - single organ dysfunction (including hemodialysis)
Grade IVb	Life-threatening complications requiring ICU management - multi-organ dysfunction
Grade V	Death of the patient

### Statistical analysis

Patient age, tumor size, length of postoperation hospital stay, blood loss during operation, and blood transfusion during operation were expressed as mean ± SD, and two-way analysis of variance was used for comparison among all the groups for parametric analysis. Nonparametric data, such as sex (male/female), location of tumor (right/left), cancer category, and complication rates were evaluated using Fisher’s exact or chi-square analyses. We used multivariable logistic regression models to estimate the association between age (<40, 40–49, 50–59, 60–69, 70–79, and ≥80 years), gender (male/female), hypertension (yes/no), diabetes (yes/no), BMI (underweight/normal/overweight/obese), tumor size, volume of blood loss during operation, and length of operation time, as well as volume of blood transfusion during operation and our primary outcomes. We specified each outcome (complication) as a binary (yes/no) variable. All statistical testing was two-sided, performed using SPSS version 19.0 for Windows (SPSS Inc., Chicago, IL, USA); *P* < 0.05 was considered statistically significant.

## Results

Among the 526 patients who had ORN, five patients were initially scheduled for LRN and converted to ORN (three cases were due to peri-renal adhesions and the other two cases were due to bleeding of the renal vein), and 12 patients were initially scheduled for OPN and converted to ORN (10 cases were due to urinary collecting system damaged during operation and the other two cases were due to bleeding of the renal wound surface). Among the 187 patients who had OPN, two patients were initially scheduled for LPN and converted to OPN (due to bleeding of the renal wound surface). Basic information for patients are shown in Table 2. No statistical significance in patient age, gender (male/female), location of tumor (right/left), blood loss or blood transfusion during operation was observed among the patients receiving LRN and ORN, nor for patients receiving LPN and OPN. Various types of renal tumors were observed, including angioleiomyolipoma, clear cell renal cell carcinoma, papillary renal cell carcinoma, chromophobe renal cell carcinoma, renal medullary carcinoma, collecting duct carcinoma, Xp11 translocation renal cell carcinoma, mucinous tubular and spindle cell carcinoma, sarcomatoid renal cell carcinoma, oncocytoma, metanephric adenoma, cystic nephroma, multilocular cystic nephroma, synovial sarcoma (monophasic), carcinoid tumor, primitive neumectodermai tumor, liposarcoma, pleomorphic sarcoma, adult rhabdomyosarcoma, squamous cell carcinoma, non-Hodgkin's lymphoma, solitary fibrous tumor, and inflammatory pseudotumor. A higher rate of angioleiomyolipoma was observed in partial nephrectomy than radical nephrectomy. Patients who received ORN had a larger tumor size (5.3 ± 2.3 cm versus 4.6 ± 1.9 cm, *P* = 0.002), and 1.6 days longer postoperation hospital stay (9.2 days versus 7.6 days, *P* < 0.001) compared with those who received LRN. Laparoscopic nephrectomy, including both LRN and LPN, had a longer operation time than open nephrectomy. The operation time of LPN was significantly 27.9 minutes longer than that for OPN (154.1 ± 141.2 minutes versus 126.2 ± 91.3 minutes, *P* = 0.034).

**Table 2:** Patient basic characteristics by surgical approach

	**Radical nephrectomy**				**Partial nephrectomy**			
	**LRN**		**ORN**		**LPN**		**OPN**	
**Patient numbers**	**614**				**229**			
	**88**		**526**		**42**		**187**	
Age (years)	56.5 ± 13.3		57.32 ± 12.4		53.2 ± 14.0		51.5 ± 13.2	
Male/female	55/33		341/185		19/23		119/68^a^	
Right/left	49/39		263/263		21/21		89/98	
Category of cancer	(n)	(%)	(n)	(%)	(n)	(%)	(n)	(%)
Angioleiomyolipoma	2	2.27	28	5.32	14	33.33	50	26.74
Malignant renal cell tumors								
Clear cell renal cell carcinoma	67	76.14	399	75.86	21	50.00	110	58.82
Papillary renal cell carcinoma	5	5.68	19	3.61	3	7.14	8	4.28
Clear cell renal cell carcinoma combined with papillary renal cell carcinoma	0	0.00	5	0.95	1	2.38	0	0.00
Chromophobe renal cell carcinoma	4	4.55	13	2.47	0	0.00	5	2.67
Renal medulary carcinoma	1	1.14	0	0.00	0	0.00	0	0.00
Collecting duct carcinoma	0	0.00	3	0.57	0	0.00	0	0.00
Xp11 translocation renal cell carcinoma	1	1.14	0	0.00	0	0.00	0	0.00
Mucinous tubular and spindle cell carcinoma	0	0.00	1	0.19	0	0.00	0	0.00
Renal cell carcinoma unclassified								
Sarcomatoid renal cell carcinoma	0	0.00	1	0.19	0	0.00	1	0.53
Renal cell carcinoma	5	5.68	33	6.27	0	0.00	6	3.21
Benign renal cell tumors								
Oncocytoma	2	2.27	6	1.14	1	2.38	2	1.07
Metanephric tumors								
Metanephric adenoma	0	0.00	0	0.00	1	2.38	0	0.00
Mixed mesenchymal and epithelial tumors								
Cystic nephroma	0	0.00	2	0.38	0	0.00	1	0.53
Multilocular cystic nephroma	0	0.00	5	0.95	0	0.00	4	2.14
Synovial sarcoma (monophasic)	0	0.00	1	0.19	0	0.00	0	0.00
Neuroendocrine tumors								
Carcinoid tumor	0	0.00	1	0.19	0	0.00	0	0.00
Primitive neumectodermai tumor	0	0.00	1	0.19	0	0.00	0	0.00
Mesenchymal tumors								
Liposarcoma	0	0.00	1	0.19	0	0.00	0	0.00
Pleomorphic sarcoma	0	0.00	1	0.19	0	0.00	0	0.00
Adult rhabdomyosarcoma	0	0.00	1	0.19	0	0.00	0	0.00
Squamous cell carcinoma	1	1.14	1	0.19	0	0.00	0	0.00
Non-Hodgkin's lymphoma	0	0.00	1	0.19	1	2.38	0	0.00
Other tumors								
Solitary fibrous tumor	0	0.00	2	0.38	0	0.00	0	0.00
Inflammatory pseudotumor	0	0.00	1	0.19	0	0.00	0	0.00
Tumor size (cm)	4.6 ± 1.9		5.3 ± 2.3^b^		3.3 ± 2.2		3.6 ± 1.4	
Length of postoperation hospital stay (days)	7.6 ± 2.4		9.2 ± 3.9^c^		8.5 ± 3.1		9.3 ± 3.8	
Length of operation time (minutes)	133.7 ± 101.4		120.3 ± 78.8		154.1 ± 141.2		126.2 ± 91.3^d^	
Blood loss during operation (ml)	235.9 ± 411.9		232.1 ± 379.5		191.1 ± 166.0		231.5 ± 222.5	
Blood transfer during operation (ml)	67.4 ± 224.6		86.4 ± 322.6		19.0 ± 86.2		36.4 ± 115.3	

^a^*P* = 0.028; ^b^*P* = 0.002; ^c^*P* < 0.001; ^d^*P* = 0.034. LPN, laparoscopic partial nephrectomy; LRN, laparoscopic radical nephrectomy; OPN, open partial nephrectomy; ORN, open radical nephrectomy.

Table 3 illustrates the overall complication rates and complication rates for each category, as well as every specific complication rate. The overall complication rate was 19.31%, 30.04%, 35.71%, and 36.36% with LRN, ORN, LPN, and OPN, respectively. The non-complication rate for ORN was significantly lower than that for LRN (odds ratio = 0.867, 95% CI 0.772 to 0.974, *P* = 0.039). Complication rates for each category and every specific complication rate were similar among radical and partial nephrectomy. Gastrointestinal complications were most common among all the postoperation complications (6.82% in LRN, 10.08% in ORN, 11.90% in LPN, and 13.90% in OPN). Two patients (0.038%) died after receiving ORN.

**Table 3:** Complication rates by surgical approach

	**Radical nephrectomy**				**Partial nephrectomy**			
	**LRN**		**ORN**		**LPN**		**OPN**	
	**n**	**%**	**n**	**%**	**n**	**%**	**n**	**%**
Total patient number	88		526		42		187	
Non-complication	71	80.68	368	69.96	27	64.29	119	63.64
Genitourinary	1	1.14	7	1.33	0	0.00	0	0.00
Acute renal failure	1	1.14	7	1.33	0	0.00	0	0.00
Wound	0	0.00	7	1.33	0	0.00	6	3.21
Infection	0	0.00	1	0.19	0	0.00	1	0.53
Subcutaneous hydrops	0	0.00	6	1.14	0	0.00	5	2.67
Infectious	1	1.14	13	2.47	0	0.00	3	1.60
Urinary tract infection	0	0.00	3	0.57	0	0.00	1	0.53
Fever of unknown origin	1	1.14	8	1.52	0	0.00	2	1.07
Sepsis	0	0.00	2	0.38	0	0.00	0	0.00
Gastrointestinal	6	6.82	53	10.08	5	11.90	26	13.90
Hiccup	0	0.00	2	0.38	0	0.00	0	0.00
Stress ulcer	0	0.00	14	2.66	2	4.76	7	3.74
Small-bowel obstruction	0	0.00	3	0.57	0	0.00	2	1.07
Constipation	3	3.41	15	2.85	1	2.38	5	2.67
Gastrointestinal bleeding	0	0.00	1	0.19	0	0.00	4	2.14
Emesis	3	3.41	13	2.47	2	4.76	6	3.21
Biliary colic	0	0.00	0	0.00	0	0.00	1	0.53
Pancreatitis	0	0.00	1	0.19	0	0.00	0	0.00
Pancreatic fistula	0	0.00	4	0.76	0	0.00	1	0.53
Cardiac	4	4.55	20	3.80	1	2.38	9	4.81
Arrhythmia	1	1.14	6	1.14	0	0.00	2	1.07
Myocardial infarction	0	0.00	2	0.38	0	0.00	0	0.00
Congestive heart failure	0	0.00	1	0.19	0	0.00	0	0.00
Hypertension	3	3.41	11	2.09	1	2.38	7	3.74
Pulmonary	2	2.27	10	1.90	4	9.52	8	4.28
Pneumonia/pneumonitis	1	1.14	4	0.76	2	4.76	2	1.07
Pneumothorax	0	0.00	1	0.19	0	0.00	2	1.07
Respiratory distress	0	0.00	1	0.19	1	2.38	1	0.53
Pleural effusion	1	1.14	2	0.38	1	2.38	3	1.60
Atelectasis	0	0.00	2	0.38	0	0.00	0	0.00
Thromboembolic	0	0.00	1	0.19	0	0.00	0	0.00
Deep venous thrombosis	0	0.00	1	0.19	0	0.00	0	0.00
Bleeding	1	1.14	11	2.09	3	7.14	7	3.74
Perinephric hematoma	0	0.00	0	0.00	1	2.38	0	0.00
Other postoperative hemorrhage	0	0.00	7	1.33	0	0.00	3	1.60
Anemia	1	1.14	4	0.76	2	4.76	4	2.14
Neurologic	0	0.00	3	0.57	1	2.38	1	0.53
Cerebrovascular event	0	0.00	1	0.19	1	2.38	1	0.53
Neuropathy	0	0.00	1	0.19	0	0.00	0	0.00
Syncope	0	0.00	1	0.19	0	0.00	0	0.00
Miscellaneous	2	2.27	33	6.27	1	2.38	8	4.28
Anaphylaxis	1	1.14	9	1.71	0	0.00	0	0.00
Chylous leak	0	0.00	6	1.14	0	0.00	0	0.00
Gout	0	0.00	1	0.19	0	0.00	2	1.07
Hyponatremia	0	0.00	2	0.38	0	0.00	1	0.53
Shock	0	0.00	0	0.00	0	0.00	1	0.53
Hypoalbuminemia	1	1.14	15	2.85	1	2.38	4	2.14
Death	0	0.00	2	0.38	0	0.00	0	0.00

LPN, laparoscopic partial nephrectomy; LRN, laparoscopic radical nephrectomy; OPN, open partial nephrectomy; ORN, open radical nephrectomy.

Table 4 shows the standardized complication rates among the four surgical approaches by using the modified CCS. Low-grade complication rates in ORN were moderately higher than that in LRN (27.19% versus 18.18%, *P* = 0.074). More Grade II complications were observed with ORN compared to LRN (odds ratio = 2.593, 95% CI 1.172 to 5.737, *P* = 0.010). No significant difference in high-grade complication rates was observed between radical and partial nephrectomy.

**Table 4:** Standardized complication rates by surgical approach

	**Radical nephrectomy**					**Partial nephrectomy**				
	**LRN**		**ORN**			**LPN**		**OPN**		
**Total number**	**88**		**526**			**42**		**187**		
	**n**	**%**	**n**	**%**	***P***	**n**	**%**	**n**	**%**	***P***
Non-complication	71	80.68	368	69.96	0.039^a^	27	64.29	119	63.64	0.973
Low grade	16	18.18	143	27.19	0.074	15	35.71	61	32.62	0.700
Grade I	10	11.36	50	9.51	0.587	4	9.52	20	10.70	1.000*
Grade II	6	6.82	93	17.68	0.010^b^	11	26.19	41	21.93	0.551
High grade	1	1.14	15	2.85	0.714*	0	0.00	7	3.74	0.355*
Grade IIIa	0	0.00	6	1.14	0.601*	0	0.00	6	3.21	0.596*
Grade IIIb	0	0.00	0	0.00	NA	0	0.00	1	0.53	1.000*
Grade IVa	1	1.14	7	1.33	1.000*	0	0.00	0	0.00	NA
Grade IVb	0	0.00	0	0.00	NA	0	0.00	0	0.00	NA
Grade V	0	0.00	2	0.38	1.000*	0	0.00	0	0.00	NA

*Fisher’s exact test. ^a^Odds ratio = 0.867, 95% CI 0.772 to 0.974; ^b^odds ratio = 2.593, 95% CI 1.172 to 5.737. LPN, laparoscopic partial nephrectomy; LRN, laparoscopic radical nephrectomy; NA, not applicable; OPN, open partial nephrectomy; ORN, open radical nephrectomy.

Univariable analyses of Grade II complications in radical nephrectomy are shown in Table 5. The association between Grade II complication rate and age, gender, location of tumor, BMI, height, weight, systolic pressure, diastolic pressure, hypertension status, fasting blood glucose level, diabetes status, tumor size, length of operation time, and blood loss during operation, as well as blood transfusion during operation were examined separately. BMI (*P* = 0.035), blood loss during operation (*P* < 0.001), and blood transfusion during operation (*P* = 0.001) were significant predictors for Grade II complications in radical nephrectomy. These three variables and those with a *P* value less than 0.500 were included in multivariable analysis. As shown in Table 5, surgical approach (LRN/ORN) (*P* = 0.036), age (*P* = 0.044), height (*P* = 0.020), systolic pressure (*P* = 0.012), fasting blood glucose level (*P* = 0.032), and blood loss during operation (*P* = 0.011) were significant predictors for Grade II complications in radical nephrectomy.

**Table 5:** Univariable and multivariable analysis of Grade II complications in laparoscopic radical nephrectomy and open radical nephrectomy

**Variable**	**Univariable**	**Multivariable**
	***P***	***P***
Surgical approach (laparoscopic/open)	0.010	0.036
Age	0.078	0.044
Gender	0.798	
Location	0.892	
Body mass index	0.035	0.230
Height	0.12	0.020
Weight	0.672	
Systolic pressure	0.052	0.012
Diastolic pressure	0.658	
Hypertension status	0.225	0.441
Fasting blood glucose level	0.253	0.032
Diabetes status	0.365	0.524
Tumor size	0.326	0.170
Length of operation time	0.522	
Blood loss during operation	<0.001	0.011
Blood transfusion during operation	0.001	0.340

## Discussion

With the increased use of laparoscopy in urological surgeries, numerous studies have documented the benefits of minimally invasive surgery. We are the first in China to compare the complication rates of laparoscopic and open nephrectomy using standardized reporting methodology. In our study, as shown in Table 3, we found that the non-complication rate with ORN was significantly lower than that with LRN (69.96% versus 80.68%, *P* = 0.039). Similar findings were reported by Tan and colleagues 12 in 2011. With respect to LPN, we did not find any significantly lower complication rate compared to OPN. This might be limited by the relatively small patient population. In addition, as shown in Table 2, patients who received ORN had significantly longer postoperation hospital stay (9.2 days versus 7.6 days, *P* < 0.001). This suggests that patients who received ORN need more postoperation recovery time. However, the complication rates for each category and every specific complication rate did not seem to be decreased either in LRN or in LPN.

In our study, increased complication rates were mainly limited to low–grade complications. Patients who received ORN had a moderately higher risk of developing low-grade complications. With further examination, we find that increased Grade II complication rate was the main factor contributing to the higher complication rate in ORN. In other words, LRN might not reduce the high-grade complication rate. Since we included patients in our study from 2006, open and laparoscopic nephrectomy technologies are well practiced and established. Furthermore, improved anesthesia technologies and postoperative monitoring also help to prevent high-grade complications. However, two patients (0.38%) died after ORN. The mortality rate was relatively low compared with other studies [12]. This was also a benefit of the improvement in the related technologies.

Using multivariable analysis, patient age contributed significantly to higher Grade II complication rates. Similar findings were also reported by Lowrance and colleagues. They found that age was significantly associated with a small increase in the risk of complications (odds ratio for 10-year increase in age 1.17, 95% CI 1.04 to 1.32, *P* = 0.009). However, in another study by Reifsnyder and colleagues 5, age was not a significant predictor for more overall complications. In both Lowrance and colleagues’ [13] and our studies, age was taken into the analysis as an ordered categorical variable rather than a continuous variable. This is more rational and clinically more meaningful than investigating how much increased risk would be present if the patient was 1 year older. It helps urologists understand the influence of age on post-nephrectomy complications and explain it to the patients.

Blood loss during the operation was another significant predictor for higher Grade II complications. Although we found that LRN did not have less blood loss during the operation than ORN, more blood loss would result in more post-nephrectomy complications. This finding was consistent with the analysis of Reifsnyder and colleagues 5 showing that more blood loss predicted more overall complications. Higher systolic pressure and fasting blood glucose level would also contribute to more Grade II complications. This suggests that hypertension and diabetes might play an important role in post-nephrectomy complications. However, neither hypertension nor diabetes status was a significant predictor in our multivariable analysis. Further study focusing on the effect of hypertension and diabetes on post-nephrectomy complications is needed.

Laparoscopic nephrectomy had a longer operation time than that of open nephrectomy, accompanied by a relatively lower complication rate. However, it could not be inferred that a longer operation time was associated with lower complication rate solely based on this. Though offering a better operation vision, laparoscopic nephrectomy could take a considerably longer time to perform than open nephrectomy. Laparoscopic techniques need relatively skilled experience and beginners usually take a longer time. Besides, laparoscopic nephrectomy requires more delicate and precise operation movements which partly contribute to the longer operation time. We cannot jump to the conclusion that a longer operation time would lead to a lower complication rate only according to superficial data. Delicate and precise operation movements not only partly result in a longer operation time, but also a lower complication rate.

Although the average tumor size in both ORN and LRN was greater than 4 cm, we observed that the tumor size in ORN was significantly larger than that in LRN (Table 2). We further examined whether a bigger tumor size might contribute to a higher complication rate using univariable and multivariable analysis. Tumor size was not a significant predictor for complication rates with radical nephrectomy. The difference in tumor size between ORN and LRN may be caused by the relatively small sample size for LRN; in addition, we did not group patients randomly. In China, National Health Insurance does not cover all surgeries. A large proportion of the cost of laparoscopic surgeries, including laparoscopic nephrectomy, is at the patient's own expense. Patients have to pay for the trocars, ultrasonic scalpel, hemolocks, and so forth. Thus we were not able to randomize the patients into a laparoscopic nephrectomy group and an open nephrectomy group. We offered both choices to the patients, and the choice was made by the patients themselves. This non-random selection has potential bias, which might contribute to the significant difference in tumor size.

We have also observed that wound complications, including wound infection and subcutaneous hydrops, only appeared in the open nephrectomy. Previous studies have reported that wound infection in laparoscopic nephrectomy is quite rare [14,15]. Open nephrectomy is characterized by a long incision (20 cm), while wound length is significantly shorter in laparoscopic nephrectomy (LRN usually has a 10-cm incision; the wound length is even shorter in LPN). Besides better wound cosmetics and less wound pain, laparoscopic nephrectomy had a lower risk of wound infection and subcutaneous hydrops because less damage was made and less wound surface was exposed.

## Conclusion

In conclusion, we found that LRN had the advantages of less Grade II complications and shorter postoperation hospital stay than ORN. There was no significant difference in complication rates between LPN and OPN. Older age and more blood loss during the operation also contribute to more Grade II complications in RN. Furthermore, hypertension and diabetes status pose potential risks for complications.

There are several limitations of this study to be considered. First, the sample size of the laparoscopic nephrectomy group was relatively small. This limitation was due to the fact that the cost of laparoscopic nephrectomy is relatively higher than open nephrectomy in China and is not all covered by National Health Insurance. A large portion of patients could not afford laparoscopic nephrectomy and chose open nephrectomy. We still enrolling subjects and further investigating the difference in complications between laparoscopic and open nephrectomy. Second, our study focused on the post-nephrectomy complications in the early stage. In our new study, we plan to prolong the follow-up and investigate complications of laparoscopic and open nephrectomy for longer periods.

## Abbreviations

BMI: body mass index; CCS: Clavien classification system; LPN: laparoscopic partial nephrectomy; LRN: laparoscopic radical nephrectomy; OPN: open partial nephrectomy; ORN: open radical nephrectomy; WHO: World Health Organization.

## Competing interests

The authors declare that they have no competing interests.

## Authors’ contributions

HWJ and QD conceived and designed the study. HX and HWJ collected data. HX analyzed the data and conducted analysis. HX wrote the manuscript. All authors read and approved the final manuscript.

## References

[B1] Siegel R Naishadham D Jemal A Cancer Statistics, 2012 CA Cancer J Clin 2012 62 10 29 10.3322/caac.20138 22237781

[B2] Clayman RV Kavoussi LR Soper NJ Dierks SM Meretyk S Darcy MD Roemer FD Pingleton ED Thomson PG Long SR Laparoscopic nephrectomy: initial case report J Urol 1991 146 278 282 1830346 10.1016/s0022-5347(17)37770-4

[B3] Dunn MD Portis AJ Shalhav AL Elbahnasy AM Heidorn C McDougall EM Clayman RV Laparoscopic versus open radical nephrectomy: a 9-year experience J Urol 2000 164 1153 1159 10.1016/S0022-5347(05)67131-5 10992356

[B4] Shuford MD McDougall EM Chang SS LaFleur BJ Smith JA Jr Cookson MS Complications of contemporary radical nephrectomy: comparison of open vs laparoscopic approach Urol Oncol 2004 22 121 126 10.1016/S1078-1439(03)00137-6 15082009

[B5] Reifsnyder JE Ramasamy R Ng CK Dipietro J Shin B Shariat SF Del Pizzo JJ Scherr DS Laparoscopic and open partial nephrectomy: complication comparison using the Clavien system JSLS 2012 16 38 44 10.4293/108680812X13291597716942 22906328 PMC3407455

[B6] Clavien PA Sanabria JR Strasberg SM Proposed classification of complications of surgery with examples of utility in cholecystectomy Surgery 1992 111 518 526 1598671

[B7] Dindo D Demartines N Clavien PA Classification of surgical complications: a new proposal with evaluation in a cohort of 6336 patients and results of a survey Ann Surg 2004 240 205 213 10.1097/01.sla.0000133083.54934.ae 15273542 PMC1360123

[B8] WHO Expert Consultation Appropriate body-mass index for Asian populations and its implications for policy and intervention strategies Lancet 2004 363 157 163 14726171 10.1016/S0140-6736(03)15268-3

[B9] Kurta JM Thompson RH Kundu S Kaag M Manion MT Herr HW Russo P Contemporary imaging of patients with a renal mass: does size on computed tomography equal pathological size? BJU Int 2009 103 24 27 18710440 10.1111/j.1464-410X.2008.07941.x PMC2634853

[B10] Lopez-Beltran A Scarpelli M Montironi R Kirkali Z WHO classification of the renal tumors of the adults Eur Urol 2004 2006 49 798 805 10.1016/j.eururo.2005.11.035 16442207

[B11] Weingart SN Iezzoni LI Davis RB Palmer RH Cahalane M Hamel MB Mukamal K Phillips RS Daves DT Banks NJ Use of administrative data to find substandard care - Vvalidation of the complications screening program Med Care 2000 38 796 806 10.1097/00005650-200008000-00004 10929992

[B12] Tan HJ Wolf JS Jr Ye Z Wei JT Miller DC Complications and failure to rescue after laparoscopic versus open radical nephrectomy J Urol 2011 186 1254 1260 10.1016/j.juro.2011.05.074 21849185

[B13] Lowrance WT Yee DS Savage C Cronin AM O’Brien MF Donat SM Vickers A Russo P Complications after radical and partial nephrectomy as a function of age J Urol 2010 183 1725 1730 10.1016/j.juro.2009.12.101 20299040 PMC4179201

[B14] Parsons JK Varkarakis I Rha KH Jarrett TW Pinto PA Kavoussi LR Complications of abdominal urologic laparoscopy: longitudinal five-year analysis Urology 2004 63 27 32 10.1016/j.urology.2003.10.003 14751341

[B15] Simon SD Castle EP Ferrigni RG Lamm DL Swanson SK Novicki DE Andrews PE Complications of laparoscopic nephrectomy: the Mayo clinic experience J Urol 2004 171 1447 1450 10.1097/01.ju.0000117942.61971.41 15017195

